# Use of non-selective B-blockers is safe in hospitalised decompensated cirrhosis patients and exerts a potential anti-inflammatory effect: Data from the ATTIRE trial

**DOI:** 10.1016/j.eclinm.2022.101716

**Published:** 2022-11-14

**Authors:** Thais Tittanegro, Louise China, Ewan Forrest, Yiannis Kallis, Stephen D. Ryder, Gavin Wright, Nick Freemantle, Alastair O'Brien

**Affiliations:** a Institute of Liver and Digestive Health, University College London, United Kingdom; b Glasgow Royal Infirmary, Glasgow, United Kingdom; c Barts and the London School of Medicine and Dentistry Queen Mary University of London, United Kingdom; d National Institute for Health Research Nottingham Biomedical Research Centre at Nottingham University Hospitals NHS Trust, and the University of Nottingham, Queens Medical Centre, Nottingham, United Kingdom; e Mid and South Essex NHS Foundation Trust, Basildon & Thurrock University Hospitals NHS Foundation Trust, The Royal Free Hospital, University College London, Kings College London, United Kingdom; f Comprehensive Clinical Trials Unit, University College London, United Kingdom

**Keywords:** Renal dysfunction, Interleukin-8, White cell count, infection

## Abstract

**Background:**

Nonselective B-blockers (NSBBs) are believed to have pleiotropic effects beyond reducing portal pressure. However, studies also report potential harm in patients hospitalized with cirrhosis and ascites. We therefore investigated whether NSBB use at ATTIRE trial entry (Albumin to prevent infection in chronic liver failure, 2016-19) was associated with increased renal or cardiovascular dysfunction, compared the incidence of infection and plasma markers of systemic inflammation, and examined mortality at 28-days, 3 and 6-months.

**Methods:**

In ATTIRE patients grouped by NSBB use at trial entry, we studied infection at baseline, hospital acquired infection and organ dysfunction during trial treatment period and mortality, with propensity score matching to account for differences in disease severity.

**Findings:**

There were no differences in renal or cardiovascular dysfunction between patients treated with NSBBs or not, during days 3–15 of hospitalization, despite elevated serum creatinine in NSBB patients at hospitalisation. Use of NSBBs was associated with a significant reduction in infection at hospitalization (*p* = 0.006), lower white cell counts throughout hospital stay (*p* < 0.001) and reduced plasma procalcitonin (*p* = 0.009) and interlukin-8 levels (*p* = 0.04) at baseline, but markers of bacterial translocation and systemic inflammation were the same in treatment groups. There was no reduction in hospital acquired infections in patients taking NSBBs and no beneficial impact on mortality at 28-days, 3 and 6-months.

**Interpretations:**

Our real-world data from a completed randomised trial show that use of NSBBs in decompensated cirrhosis patients is safe during hospitalisation. We also show a potential anti-inflammatory role for NSBBs which may be mediated by a downregulation of IL-8 induced leucocytosis, that was associated with reduced infection at baseline but not a survival benefit.

**Funding:**

10.13039/100010269Wellcome Trust and 10.13039/501100000276Department of Health and Social Care.

**Research in context**

**Evidence before this study**

Non-selective B-blockers (NSBBs) are used to prevent bleeding from gastroesophageal bleeding and studies also support other beneficial effects, such as reducing risk of infection in cirrhosis. However, there remains concern over the safety of NSBBs in hospitalised decompensated cirrhosis patients, in particular regarding renal dysfunction.

**Added value of this study**

We show that the use of NSBBs at hospitalisation was safe with no increased incidence of subsequent renal, cardiovascular other organ dysfunction during hospital stay compared to patients not prescribed. NSBBs exhibit a potential anti-inflammatory effect with use associated with reduced white cell count, procalcitonin, plasma interleukin-8 levels, and incidence of infection at hospitalization. However, we observed no improvement in rates of hospital acquired infection nor mortality benefit at 28-days, 3 and 6-months for patients taking NSBBs compared to those not.

**Implications of all the available evidence**

Judicious use of NSBBs is safe in patients hospitalized with decompensated cirrhosis. NSBBs appear to have anti-inflammatory properties in decompensated cirrhosis but further studies are required to identify patients that may derive the most benefit, before advocating a change in practise.

## Introduction

The efficacy of non-selective Beta-Blockers (NSBBs) to prevent bleeding from oesophagogastric varices and portal hypertensive gastropathy (PHG) has been demonstrated in randomised controlled trials (RCTs).^1^^,^^2^ However, some studies in patients with ascites or refractory ascites have suggested NSBBs may cause harm by increasing renal dysfunction^3^, ^4^, ^5^, ^6^, ^7^ and a “therapeutic window” beyond which NSBBs may become detrimental has been proposed.^8^ These data have led to current recommendations in patients with ascites, that NSBBs should be dose-reduced or discontinued if persistently low blood pressure (systolic blood pressure <90 mmHg or mean arterial pressure <65 mmHg) and/or Hepato-renal syndrome-Acute Kidney injury (HRS-AKI) and restarted or dose increased once patient has improved.^9^

Other studies suggest NSBBs may have pleiotropic effects beyond reducing portal pressure with evidence that they prevent decompensation events, and several papers support increased survival independent of bleeding events.^10^, ^11^, ^12^ NSBBs may reduce bacterial translocation^13^^,^^14^ and an association between NSBB use and decreased white cell count has been demonstrated,^15^, ^16^, ^17^ suggesting NSBBs may modulate systemic inflammation with a meta-analysis indicating use was associated with a reduced risk of spontaneous bacterial peritonitis (SBP).^11^ In line with these findings, the role of NSBB treatment to prevent further (non-rebleeding) decompensation events in decompensated patients has been highlighted as a research priority.^9^

Therefore, we performed a retrospective data analysis to investigate whether the use of B-blockers at ATTIRE trial entry (Albumin to prevent infection in chronic liver failure^18^) was associated with increased renal or cardiovascular dysfunction during hospitalization. We also compared the incidence of infection, and other organ dysfunction during the trial, plasma markers of systemic inflammation and mortality at 28-days, 3 and 6-months from ATTIRE randomisation in patients treated with or without NSBBs. Finally, as carvedilol has intrinsic anti-alpha adrenergic vasodilatory effects that contribute to its greater portal pressure reducing effect compared to propranolol, we compared outcomes between patients taking these two NSBBs.^19^

## Methods

### ATTIRE trial

ATTIRE was a neutral trial of targeted albumin infusions versus standard care involving 777 hospitalized decompensated cirrhosis patients from 35 hospitals across England, Wales and Scotland (2016–2019). See Supplementary Fig. S1 for details.

### Primary analyses: the effect of use of NSBBs at ATTIRE trial entry on renal and cardiovascular dysfunction in patients hospitalized with decompensated cirrhosis

We compared baseline characteristics and incidence of renal and cardiovascular dysfunction on days 3–15 of the trial between patients prescribed NSBBs or not at baseline (trial entry). We compared daily systolic and diastolic blood pressure (lowest daily paired systolic blood pressure recordings) and daily heart rate (highest and lowest over 24 h) values during the trial in patients prescribed NSBBs or not.

**Propensity Scoring to further examine** use of NSBBs at ATTIRE trial entry on renal and cardiovascular dysfunction in patients hospitalized with decompensated cirrhosis: This was used to account for baseline differences in disease severity, when numbers were adequate to provide a robust basis for estimation, defined prospectively as ≥50. We calculated a propensity score for each subject, using the fitted value on the logit scale from a logistic regression model which included baseline use of antibiotics, suspected variceal bleed (categorized as suspected because case report form often completed prior to endoscopy as patients could be enrolled at hospitalization), new-onset/worsening ascites, hepatic encephalopathy (HE), gender, age, Model for end stage liver disease - MELD score, serum albumin, creatinine, white cell count (WCC), C-reactive protein (CRP), and randomized group. We included WCC and CRP in the model to account for increased systemic inflammation in patients with decompensated cirrhosis which has been shown to contribute significantly to clinical outcomes.^20^ Explanatory variables in the baseline model were modified until an adequate match was achieved between cases and controls assessed by relevant standardized mean differences. Cases and controls were matched on propensity scores using a ‘greedy’ nearest neighbor matching procedure without replacement, with caliper width of 0.01 on the logit scale. Once adequate matching had been achieved, the matched data set was locked before proceeding to preplanned outcome analyses.^21^^,^^22^ We performed supportive PS analyses with tigher caliper (0.001) that showed no differences.

### Secondary analyses

#### The effect of NSBBs at ATTIRE trial entry on baseline infection diagnosis and systemic inflammation

We compared the incidence of infection, baseline (and daily values during trial) WCC and C-reactive protein (CRP) and baseline plasma markers of bacterial translocation between patients taking NSBBs or not (Endotoxin binding protein (LBP) and soluble CD14 (sCD14), systemic inflammation (Tumour Necrosis Factor (TNF) and Interleukin-6 (IL-6)), infection, Procalcitonin (PCT) and the neutrophil associated chemokine Interleukin-8 (IL8)).

#### Validation of infection diagnosis

As infection diagnosis was made clinically at baseline and we did not record microbiology data, we compared age, MELD, creatinine, CRP, WCC and mortality between all patients diagnosed with or without infection at baseline.

#### The effect of NSBBs on hospital acquired infection (HAI), systemic inflammation and other organ dysfunction during the ATTIRE trial treatment period and mortality at 28-days, 3 and 6-months

We investigated HAIs, brain and respiratory dysfunction on days 3–15 of the trial between patients prescribed NSBBs or not at baseline (trial entry) during the trial. We investigated mortality at 28-days, 3 and 6-months during trial follow-up between patients prescribed NSBBs or not at baseline, choosing a categorial analysis approach rather than Kaplan Meier as we were examining short-term outcomes and all patients had the same follow-up times. Propensity score matching was performed to account for baseline differences in disease severity, as above.

##### Sample processing

Blood samples were obtained from patients in both study arms prior to trial-related albumin infusions being administered. Data were taken from our published dataset in which 142 patients' samples (n = 71 in targeted HAS arm, n = 71 in standard of care arm) were blindly analysed.^23^ All patients selected were enrolled in the trial for at least 5 days. For this paper we divided patients into those taking NSBBs (n = 23) or not (n = 119) at baseline but did not subdivide into treatment groups as samples were taken at study recruitment prior to albumin treatment. We did not examine the plasma inflammatory markers after baseline in view of the potential confounding effect of targeted albumin therapy,^24^ although we previously found no effect of albumin on these overall.^23^ LBP, sCD14, TNF, IL-6, IL-8 and PCT evaluation was undertaken via Luminex assay (R&D Systems, Minneapolis, MN) according to the manufacturer's instructions – see Supplementary material and Supplementary Table 1 for details.

#### Use of carvedilol or propranolol and patients with NSBBs stopped during hospitalisation

We compared baseline characteristics and clinical outcomes in patients taking carvedilol or propranolol at baseline. As patients in which NSBBs were stopped during admission might represent a group with increased adverse events we examined patients in which NSBBs were stopped within 5 days of trial entry separately.

### Data collection

Data were collected daily until discharge, death, medically fit for discharge or day 15 and mortality data at 28-days, 3 and 6-months from trial entry.

B-blocker use was extracted from the concomitant medication (ConMed) case report forms (CRFs). We searched for Propranolol, Carvedilol, Nadolol and Timolol, with only Propranolol and Carvedilol found. Data included name, dose, start/stop date for all medications during trial from drug charts and were inputted into the ATTIRE database at UCL Comprehensive Clinical Trials Unit.

ATTIRE defined renal dysfunction during trial treatment period, defined as serum creatinine increase ≥50% from randomisation, or patient initiated on renal replacement, or rise in creatinine ≥26.5 μmol/L within 48 h. Daily incidence of respiratory, circulatory and cerebral dysfunction (grade 3 or 4 hepatic encephalopathy) during the treatment period (based on modified components of the Chronic liver failure-sequential organ failure assessment (CLIFSOFA) score,^25^ Supplementary Table 2) as well as pulse and blood pressure were recorded. Infection was according to attending clinician's diagnosis and sites were then asked to complete infection CRFs with supporting clinical, biochemical, microbiological and radiological data. These were blindly scrutinized by a panel of 3 physicians to categorize information provided as making infection diagnosis “likely” or “unlikely”. Blood test results were taken from values obtained at each hospital site.

### Statistical analysis

A statistical analysis plan, prior to analyses, was approved by all authors. All authors vouch for completeness and accuracy of data. Confidence intervals were not adjusted for multiple comparisons and should not be used to infer definitive treatment effects. Microsoft Excel was used for extraction of data from ATTIRE databases, producing tables and graphs.

IBM SPSS – Version 27 was used for bivariate tests of statistical significance (T-tests for continuous variables and Fishers exact or Chi-squared tests for categorical variables). Other analyses performed using SAS software, version 9.4 (SAS Institute; Carey NC).

### Ethics

The ATTIRE trial was approved by the London–Brent Research Ethics Committee (ref:15/LO/0104) and the Medicines and Healthcare Products Regulatory Agency (MHRA, ref: 20363/0350/001-0001). Written informed consent was obtained from the patients. For incapacitated patients, a legal representative provided written informed consent until the patient regained capacity.

### Role of the funder

This work was funded by the Health Innovation Challenge fund awarded to Dr O'Brien 10.13039/100010269Wellcome Trust and 10.13039/501100000276Department of Health and Social Care) HICF reference HICF-R8-439, WT grant number WT102568. This funding source had no role in the design of this study and will not have any role during its execution, analyses, interpretation of the data, or decision to submit results.

## Results

### Comparison of patients taking non-selective B-blockers or not at trial entry

#### Baseline characteristics

At randomisation, there were 139 (17.9%) identified patients taking B-blockers out of 777 randomised patients, with 69 prescribed Carvedilol, median dose 6.25 mg (IQR 6.25–12.5) once a day, and 70 Propranolol, median dose 40 mg (IQR 40–80) once a day. Patients prescribed NSBBs at baseline had similar age, gender, presence of ascites, MELD score serum albumin and CRP to those not, but significantly increased incidence of suspected variceal bleeds and serum creatinine and significantly reduced WCC (Table 1). In patients diagnosed with alcoholic hepatitis at baseline, a similar number were taking NSBBs (28/139, 20.1%) as not (163/638, 25.5%), p = 0.18.

**Table 1:** Overall patient characteristics and clinical outcomes.

	No NSBB	%/SD	Baseline NSBB	%/SD	*P* value	Standardised mean differences (SMD)
Number	638	82.1%	139	17.9%	n/a	n/a
Albumin Treatment	312	50.0%	68	48.9%	1.000	0.04
Mean age (yrs)	53.9	10.9	53.6	9.4	0.74	0.03
Male	448	70.3%	101	72.7%	0.61	0.05
Suspected Variceal Bleed	86	13.7%	29	21.2%	0.034∗	0.20
Ascites	423	66.7%	94	67.6%	0.92	0.02
Hepatic Encephalopathy	127	20.2%	22	15.8%	0.29	0.11
Baseline antibiotic use	341	53.7%	67	48.2%	0.26	0.11
MELD Score	19.9	6.2	19.0	6.5	0.13	0.14
Serum Albumin (g/L)	23.1	3.8	23.5	3.5	0.32	0.09
Creatinine (mmol/L)	81.2	57.2	92.7	61.4	0.03∗	0.19
WCC (x10^9^/L)	9.3	5.4	7.0	3.7	<0.0001∗	0.50
CRP (mg/L)	38.7	40.9	36.0	76.9	0.71	0.04
Clinical outcomes						
Diagnosis of infection at randomization	186	29.2	25	18.0	0.006∗	
Incidence of new infection	128	20.1	22	15.8	0.29	
Incidence of new renal dysfunction	86	13.5	11	7.9	0.088	
Incidence of new cerebral dysfunction	34	5.3	10	7.2	0.42	
Incidence of new circulatory dysfunction	38	6.0	8	5.8	1.00	
Incidence of new respiratory dysfunction	105	16.5	20	14.4	0.61	
28-day mortality	98	15.4	17	12.2	0.43	
90-day mortality	159	25.0	26	18.7	0.13	
180-day mortality	210	33.0	41	29.5	0.48	

∗Denotes significant difference p < 0.05. Data are mean unless stated.

#### Use of NSBBs was not associated with renal or cardiovascular dysfunction during the ATTIRE trial treatment period

**Fig. 1:**
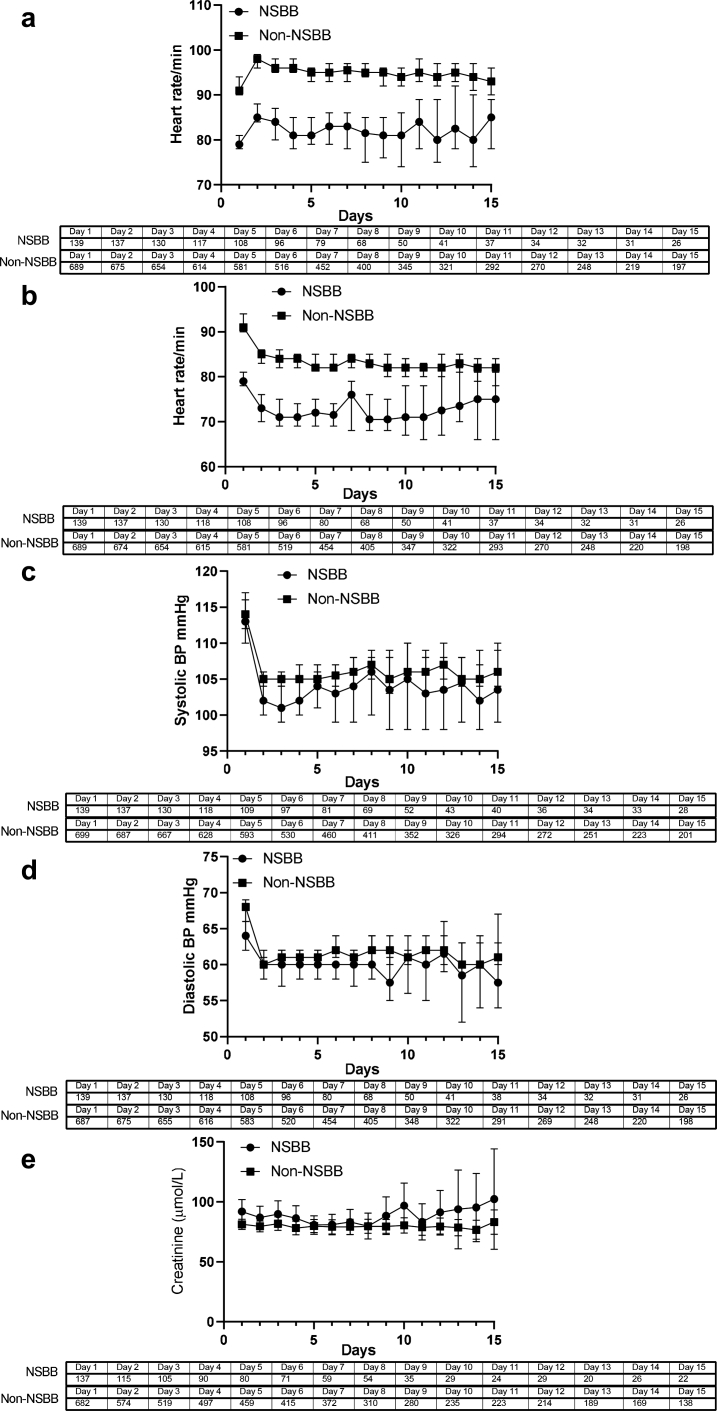
Daily heart rate (highest (a) and lowest (b)), lowest daily paired systolic (c) and diastolic (d) blood pressure readings and daily serum creatinine (e) in patients taking NSBBs or not at ATTIRE trial entry (median and 95% CI). The daily heart rates (highest and lowest) were significantly lower in the NSBB group throughout the trial treatment period) (p < 0.0001 days 1–12 and days 13–14 p < 0.005 and day 15 p < 0.05) but there were no significant differences in blood pressure nor differences in serum creatinine values throughout the trial treatment period between treatment groups.

There were no differences between the incidence of renal dysfunction (13% in non-NSBBs and 7% in NSBBs, *p* = 0.088) and circulatory dysfunction (6% in non-NSBBs vs 5.8% in NSBBs, *p* = 1) between days 3–15 of the trial treatment period between patients taking NSBBs or not at trial entry, baseline (Table 1).The daily heart rates (highest and lowest) were significantly lower in the NSBB group throughout the trial treatment period) (*p* < 0.0001 days 1–12 and days 13–14 *p* < 0.005 and day 15 p < 0.05) but there were no significant differences in blood pressure nor differences in serum creatinine values throughout the trial treatment period between treatment groups (Fig. 1 a–e).

#### Propensity score matched analysis

Non-NSBB and NSBB patients were not evenly matched at baseline with NSBB patients having increased incidence of variceal bleed and increased creatinine, and so we undertook propensity score matching to account for these differences between groups. Propensity scores (PS) were calculated, and 252 patients were matched (126 prescribed NSBBs matched individually with 126 not). This was successful with no significant differences between groups for baseline characteristics and low standardised mean differences between matched cohorts (Table 2). Analyses showed no differences in renal or circulatory dysfunction between groups, with matched outcomes risk differences also shown in Supplementary Table 4. We present the matched and unmatched continuous variables we have in the PS model, with both t test 95% confidence intervals (CIs) and bootstrapped 95% CIs (Supplementary Table 3) and the PS model (Supplementary Table 4).

**Table 2:** Matched patient characteristics and clinical outcomes and matched outcomes risk differences and 95% CI.

	No NSBB	%/SD	Baseline NSBB	%/SD	*P* value	SMD
Number	126	50%	126	50%	1.00	0.0
Albumin Treatment	64	50.8%	59	46.8%	0.61	0.12
Mean age (yrs)	55.3	11.7	53.5	9.1	0.17	0.17
Male	96	76.2%	93	73.8%	0.77	0.06
Suspected Variceal Bleed	21	16.7%	24	19.1%	0.74	0.06
Ascites	87	69.1%	86	68.3%	1.00	0.02
Hepatic Encephalopathy	24	19.1%	21	16.7%	0.74	0.06
Baseline antibiotic use	65	51.6%	60	47.6%	0.61	0.07
MELD Score	18.2	5.0	18.9	5.7	0.37	0.11
Serum Albumin (g/L)	23.4	3.9	23.4	3.6	0.92	0.01
Creatinine (mmol/L)	83.2	42.1	87.3	49.2	0.48	0.09
WCC (x10^9^/L) (median)	6.9	3.3	7.0	3.6	0.77	0.04
CRP (mg/L) (median)	30.2	28.5	37.1	80.0	0.39	0.12

Clinical outcomes						
	No NSBB	%	Baseline NSBB	%	*P* value	
Incidence of new infection	21	16.7	21	16.7	1.00	n/a
Incidence of new renal dysfunction	19	15.1	11	8.8	0.17	n/a
Incidence of new cerebral dysfunction	4	3.2	10	7.9	0.17	n/a
Incidence of new circulatory dysfunction	7	5.6	7	5.6	1.00	n/a
Incidence of new respiratory dysfunction	21	16.7	19	15.1	0.86	n/a
28-day mortality	15	6.0	16	6.4	1.00	n/a
90-day mortality	23	18.3	24	19.1	1.00	n/a
180-day mortality	33	26.2	37	29.4	0.67	n/a

Risk differences for matched outcomes			
**Outcome**	Risk difference	Lower 95% CI	Upper 95% CI
Incidence of new infection	0.0000	−0.0920	0.0920
Incidence of renal dysfunction	−0.0635	−0.1431	0.0161
Incidence of new cerebral dysfunction	0.0476	−0.0086	0.1039
Incidence of new circulatory dysfunction	0.0000	−0.0566	0.0566
Incidence of new respiratory dysfunction	−0.0159	−0.1061	0.0743
28-day mortality	0.0079	−0.0732	0.0890
90-day mortality	0.0079	−0.0882	0.1041
180-day mortality	0.0317	−0.0788	0.1423

Data are mean unless stated.

### Subgroup analyses

#### Use of NSBBs at ATTIRE trial entry was associated with reduced baseline infection diagnosis, serum white cell count and Interleukin-8

**Fig. 2:**
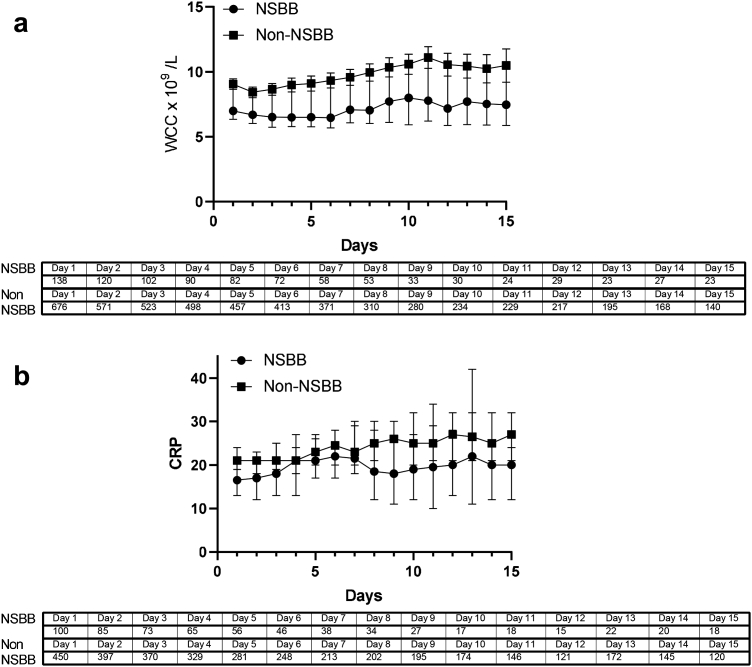
Daily WCC (a) and CRP (b) in NSBB and non-NSBB taking patients (median and 95% CI) throughout ATTIRE trial treatment period. Daily WCC values were significantly lower in NSBB patients between days 1–13 (p < 0.001 from baseline to day 6 and p < 0.05 from day 7–13, no differences on day 14 or 15).

The incidence of clinician diagnosis of infection at baseline was significantly lower in the NSBB group (18%) compared to non-NSBB (29.2%, *p* = 0.006), as were serum white cell count (WCC) values (Tables 1 and 2). We found that median PCT and IL-8 values were significantly lower in patients taking NSBBs at baseline (*p* = 0.009 and *p* = 0.04, Table 3). Serum white cell count values remained significantly lower in patients taking NSBBs throughout the trial treatment period (p < 0.001 from baseline to day 6 and p < 0.05 from day 7–13, no differences on day 14 or 15) (Fig. 2a).

**Table 3:** Baseline plasma inflammatory profile in survivors and non-survivors at 3 months post trial entry.

Plasma inflammatory mediator	No-NSBB Median (CI) (n = 119)	Baseline NSBB Median (CI) (n = 23)	*P* value
sCD14 (ng/ml)	2380 (1620–3980)	2080 (1100–101000)	0.99
LBP (ng/ml)	1910 (1630–2980)	1740 (1300–2760)	0.64
IL-6 (pg/ml)	12.6 (10.3–15.2)	11.8 (6–22.5)	0.66
TNF (pg/ml)	3.8 (3.4–4.5)	4.2 (2.4–5.6)	0.62
Procalcitonin (ng/ml)	162 (128.5–213.8)	78.8 (157.3–54.6)	0.009∗
IL-8 (pg/ml)	96 (57.8–135.2)	23 (11.6–166.0)	0.04∗

Median values were measured at baseline (data not distributed normally) and Mann–Whitney unpaired t-test used to compare groups, ∗denotes significant difference p < 0.05.

There were no significant differences between patients taking NSBBs or not for baseline plasma markers of Bacterial Translocation (LBP and sCD14) and systemic inflammation (TNF and IL-6) (Table 3). Neither were there any significant differences in CRP values throughout the trial between treatment groups (Fig. 2b).

#### Validation of clinical infection diagnosis

Patients diagnosed by their clinical team with infection at baseline had significantly increased CRP and WCCs (*p* < 0.0001) and double the mortality rates *(p* = 0.001) but similar MELD scores when compared to those not diagnosed with infection (Supplementary Table 5).

#### Use of NSBBs during hospitalisation had no effect on hospital acquired infection (HAI) or other organ dysfunction during the trial treatment period nor mortality at 28-days, 3 and 6-months

There were no differences in development of hospital acquired infections (HAIs) during the trial, nor brain and respiratory dysfunction on days 3–15 of the trial between patients prescribed NSBBs or not at baseline, with results confirmed by propensity score matching (Table 2). Finally, there were no differences seen between groups for 28 days, 3 and 6-months mortality during trial follow-up between patients prescribed NSBBs or not at baseline with results again confirmed using propensity score matching (Table 2).

#### Use of carvedilol or propranolol and patients with NSBBs stopped during hospitalisation

There were no differences between baseline characteristics, heart rate, blood pressure or serum creatinine during hospitalisation when patients taking carvedilol or propranolol at baseline were compared. There were also no differences between the incidence of HAI or renal dysfunction and mortality rates between groups (Supplementary Table 6 and Supplementary Fig. S2).

NSBBs were stopped in 29 patients (20.8%) within 5 days of trial entry, of which 5 patients died and 5 were discharged during this period. None of these 29 patients were documented as restarting on NSBBs at discharge, the other 110 patients were taking NSBBs at discharge. There were no significant differences between groups for baseline characteristics. The highest heart rate recorded was significantly higher (*p* ≤ 0.0001) and the lowest significantly lower (*p* < 0.05–0.001) on days 2–5 in patients that had NSBBs stopped within 5 days. Blood pressure, serum creatinine (Supplementary Fig. S3) and the incidence of renal dysfunction and HAI during hospitalisation were similar in both groups. The incidence of 28-day mortality was significantly higher in those in which NSBBs were stopped, but 3 and 6-month mortality was similar (Supplementary Table 7).

## Discussion

Our analyses demonstrate that the use of non-selective B-blockers (NSBBs) at baseline in hospitalised decompensated cirrhosis patients is safe with no increased renal or cardiovascular dysfunction observed during days 3–15 of the ATTIRE trial, despite elevated serum creatinine at hospitalisation. These data also support an anti-infective role at hospitalisation for NSBBs in decompensated cirrhosis that was associated with a reduction in circulating IL-8 and white cell count. However, there was no reduction in hospital acquired infections (HAIs) in patients taking NSBBs and no beneficial impact on mortality at 28-days, 3 and 6-months following trial enrolment.

Overall, the use of NSBBs, with median doses of 40 mg Propranolol and 6.25 mg Carvedilol per day, led to an expected 10% reduction in heart rate but no increased renal or cardiovascular dysfunction, nor increased grade 3/4 hepatic encephalopathy. NSBBs were discontinued in relatively few patients, 20.8% within 5 days of trial entry, although one-third of these had either died or been discharged. These patients had no significant differences in clinical characteristics at baseline to those in which NSBBs were continued and the only clinical feature measure that separated those that stopped or continued NSBBs was heart rate. Given the established benefits of NSBBs to prevent variceal bleeding,^26^ these data strongly advocate for use in this cohort and data were very similar for patients taking either propranolol or carvedilol. Although there was a trend for reduced renal dysfunction in those taking NSBBs, given the confidence intervals crossed one we believe our data should be interpreted as excluding a difference between groups and that use was safe. However, serum creatinine was significantly higher at baseline in patients taking NSBBs and evidently these drugs must be monitored carefully in patients with decompensated cirrhosis.

Use of NSBBs was associated with a significant reduction in serum WCC at baseline that was maintained throughout hospitalisation and a reduced incidence of infection at hospitalization despite NSBB and non-NSBB patients having similar MELD scores. This potential anti-infective role for NSBBs was supported by the significantly reduced procalcitonin (PCT) at baseline in those taking NSBBs. However, there were no differences in CRP values between groups beyond baseline during the trial. We did not collect data on infection type at hospitalisation and diagnosis was made clinically rather than microbiologically. However, analyses showed overall patients diagnosed with infection had significantly elevated WCC and CRP despite similar MELD scores compared to those not diagnosed and twice the in-hospital mortality rate, which support the likely validity of this diagnosis. Despite the lower incidence of infection diagnosis, the baseline use of antibiotics was similar between groups (53% in non-NSBBs vs 48% in NSBBs, *p* = 0.26), which is likely to have been secondary to use of antibiotics to treat variceal bleed patients, as this was twice as common in those taking NSBBs at baseline. Previous studies support an anti-infective effect in certain groups of cirrhosis patients taking B-Blockers,^27^ but not all,^28^ and at least two large studies have reported a reduction in WCC in cirrhosis patients taking NSBBs.^15^^,^^16^ Our laboratory analyses do not support that the reduced WCC was associated with reduced bacterial translocation in NSBB patients, as LBP and sCD14 values were no different, and there were no differences in the systemic inflammation markers TNF or IL-6. However, IL8 was significantly reduced in patients taking NSBBs at baseline. Systemic administration of IL-8 induces a rapid mobilization of progenitors from the bone marrow and causes^29^ leucocytosis.^30^ Propranolol has been shown to inhibit the stimulatory effect of dopamine on the production of IL-8 from keratinocytes^31^ and propranolol treatment led to decreased interleukin-8 levels in alveolar fluid in a rat model of passive cigarette exposure.^32^ Early use of beta-blockers prevented excessive inflammation after distal type acute aortic aneurysm dissection (AAD)^33^ and it has been shown that serum IL-8 levels are increased in patients with AAD.^34^ Marked elevation of IL-8, is associated with increased severity and poor prognosis in alcoholic hepatitis,^35^ and 90% of ATTIRE patients had alcohol-induced liver cirrhosis. We hypothesise that NSBBs exert an anti-inflammatory effect via downregulation of circulating IL-8 production leading to reduced WCC that is protective against infection in decompensated cirrhosis.

However, NSBB use was not associated with a reduction in hospital acquired infection nor any improvement in mortality at 28-days, 3 and 6-months with results confirmed by propensity score matching. These patients may have had too advanced liver disease to benefit from this anti-inflammatory effect, with a median MELD score of 20. A study in ACLF suggested a 28-day mortality benefit with NSBB, but NSBB patients had lower ACLF scores at baseline, alcohol aetiology was less common and there was no long-term benefit.^15^ In contrast, studies in patients with less severe disease have shown mortality benefit^10^, ^11^, ^12^ and there may be a therapeutic window for disease severity in which this anti-inflammatory effect of NSBBs could prevent infection and hospitalisation leading to improved outcomes.

ATTIRE was a large national trial and therefore NSBB use is likely to represent UK clinical practice and the neutral outcome for albumin enabled pooling of all data. We performed propensity score matching to account for baseline differences, but this was not a randomised comparison of B-Blockers to non-use, and despite careful but not perfect matching we cannot exclude the potential for confounding by indication. It is anticipated that the two large scale NSBB trials, CALIBRE^36^ and BOPP (ClinicalTrials.gov Identifier: NCT03776955), currently recruiting in the UK will provide additional information, although these include patients with less severe liver disease than ATTIRE. We have assumed patients taking NSBBs at randomisation were doing so prior to hospitalisation and it is possible that in some this was commenced at admission, although as patients were randomised on average on day 2 of hospitalisation, this would be unlikely as standard care would be to start NSBBs at a later stage, when patients had stabilised. We also have no data on when the patients started taking NSBBs. The term suspected variceal bleed was included on the ATTIRE case report forms as we did not collect corroborative evidence such as endoscopy reports and it is likely that several of these patients did not have significant variceal bleeds. Neither did we did not collect data regarding incidence of variceal bleed during hospitalization. Patients anticipated to have a short hospital stay were not included in ATTIRE, the numbers of patients admitted to intensive care were low and those with baseline organ dysfunction relatively so, and there were low numbers of patients with non-alcohol aetiology; therefore, our results cannot necessarily be extrapolated to these cohorts. We performed multiple tests and for some there were low numbers. Only 4 patients were transplanted during 6-month follow-up, and we did not collect data on transplant listing, ongoing alcohol consumption, or hospital readmissions after discharge. We do not have data on medications prescribed for patients after they had left hospital to inform whether NSBBs were started or stopped following discharge. Finally, it is possible that the NSBB effect of lowering WCC may have made clinicians less likely to make an infection diagnosis at hospitalization, which could have led to us overestimating the apparent anti-infective effect of NSBBs.

Given the established indications for NSBBs to prevent variceal bleeding, these real-world data from a completed randomised trial provide evidence that use in patients with decompensated cirrhosis is safe during hospitalisation. Importantly, our data also support a potential anti-inflammatory role for NSBBs which may be mediated by a downregulation of IL-8 induced leucocytosis, that was associated with reduced infection at baseline but not a survival benefit.

## Contributors

Databases were created by LC and TT and verified by AOB and NF. TT, AOB and NF performed statistical analyses. AOB wrote manuscript first draft, with contributions from all authors. All authors read and approved the final version of the manuscript.

## Data sharing statement

Data sharing can be made available upon reasonable request.

## Declaration of interests

There are no conflicts of interest.
