# Supplementary material for: Use of non-selective B-blockers is safe in hospitalised decompensated cirrhosis patients and exerts a potential anti-inflammatory effect: Data from the ATTIRE trial
Source: eClinicalMedicine. 2022 Nov 14;55:101716. doi: 10.1016/j.eclinm.2022.101716 (PMC9672423; doi:10.1016/j.eclinm.2022.101716)
Supplement: Supplementary File S1 [file mmc1.docx]

**ATTIRE Trial Investigators**

**Independent Data Monitoring Group:** Professor Dominique Valla (CHAIR), Tim Clayton and Professor Vipul Jairath.

**Data centre at University College Comprehensive Clinical Trials Unit (UCL CCTU):** Kate Bennett, Scott Bevan, James Blackstone, Kashfia Chowdhury, Zainib Shabir and Simon Skene.

**Trial Steering Committee:** Professor Stephen J. Brett (CHAIR), John Crookenden (Patient Representative), Professor Shahid A. Khan, Brennan Kahan, Professor Graeme Alexander, Professor Humphrey Hodgson and Professor Mike Murphy.

**Trial Management Group:** Dr Louise China, Dr Ewan H Forrest, Dr Yiannis Kallis, Jim Portal, Professor Stephen Ryder and Dr Gavin Wright.

**UCL CCTU:** Dr Ana Arbeloa del Moral, James Blackstone, Kashfia Chowdhury, Dr Ana Carolina Estevao, Rosie Hamilton, Ms Khadra Mohamoud and Dr Nicola Muirhead.

**Research Steering Committee:** Professor Mauro Bernardi (CHAIR), Paula Milton (Department of Health and Social Care representative) and Nicola Shepherd (Wellcome Trust representative).

**Microbiology and Adverse Event Review Panel:** Dr Indran Balakrishnan, Dr Mark McPhail, Dr Brian Hogan and Dr Jane Abbott.

**ATTIRE Site Investigators**

Professor Aftab Ala, Dr Richard Aspinall, Dr Andrew Austin, Dr C Lye Ch'ng, Dr Jeremy Cobbold, Dr Lynsey Corless, Dr Alexandra Daley, Professor Matthew Cramp, Dr Ahmed Elsharkawy, Dr Alex Evans, Dr Shaun Greer, Dr Mathis Heydtmann, Dr Coral Hollywood, Dr Peter Isaacs, Professor Rajiv Jalan, Dr Yiannis Kallis, Dr Richard Keld, Dr Andrew King, Dr Stuart McPherson, Dr Judith Morris, Professor Jane Metcalf, Dr Richard Parker, Dr Janisha Patel, Dr Francisco Porraz-Perez, Dr Praveen Rajasekhar, Dr John Ramage, Dr Paul Richardson, Dr Dariush Sadigh, Dr Deepak Suri, Dr Esther Unit, Professor Sumita Verma and Dr Earl Williams.

**ATTIRE Clinical Trial Sites**

Basildon, Basingstoke, Berkshire, Birmingham, Blackpool, Bournemouth, Bristol, Brighton, Coventry, Derby, Durham, Glasgow RI, Glasgow QE, Glasgow RA, Gloucestershire, Heartlands, Hull, Leeds, Liverpool, Manchester, Newcastle, North Tees, North Tyneside, Nottingham, Oxford, Plymouth, Portsmouth, Royal Free, Royal London, South Tyneside, Southampton, Surrey, Swansea, Whittington and Wigan.

## Supplementary Figure 1. ATTIRE patient recruitment and treatment protocol

**Supplementary Figure 2.** Daily heart rate (highest **(a)** and lowest **(b)**), lowest daily paired systolic **(c)** and diastolic **(d)** blood pressure readings and daily serum creatinine **(e)** in patients taking carvedilol or propranolol at ATTIRE trial entry (median and 95% CI).

**a**

**b**

**c**

**d**

**e**

**Supplementary Figure 3.**  Daily heart rate (highest **(a)** and lowest **(b)**), lowest daily paired systolic **(c)** and diastolic **(d)** blood pressure readings and daily serum creatinine **(e)** in patients taking NSBBs at ATTIRE trial entry that were stopped within 5 days or continued. The highest heart rate recorded was significantly higher (*p*<0.0001) and the lowest significantly lower (*p*<0.05-0.001) on days 2-5 in patients that had NSBBs stopped within 5 days. There were no differences for daily blood pressure or creatinine readings.

**d**

**c**

**e**

**a**

**b**

**Supplementary Methods**

Samples were taken using 9mL lithium heparin tubes that were labelled with the patient’s trial ID and day of sample collection and transferred to the site’s hospital laboratories and spun at 1300x g at 20°C. The plasma layer was removed and frozen at -80°C in 2mL cryovials with the corresponding trial identifier. Samples were collected from patients at 33 UK hospital sites. They were transferred to University College London at the end of the recruitment period in 2019. All analyses were conducted after first sample thaw.

We used a bead based multiplex assay that allowed accurate, concurrent measurement of multiple analytes in a small volume of sample. Briefly, after defrosting, samples were centrifuged at 16,000g for 6 minutes and then diluted in calibrator diluent RD6-52 (1:2 for all analytes apart from sCD14 and LBP in which assays plasma was diluted to 1:200). Standards were made up as per the specific product sheet and diluted 1:3 serially to produce a standard curve with the range of detection. Samples and standards were plated using the supplied opaque plate and the microparticle cocktail was added as per instruction. The plate was then sealed with foil and left overnight (14-16 hours) at 4°C on an orbital shaker at 900 rpm. Plates were washed with the addition of a plate magnet and antibody cocktail for the same analytes was added with the plate left on the orbital shaker at 900 rpm for 1 hour at room temperature. Plates were washed again, with the use of a magnetic plate, and streptavidin-phycoerythrin conjugate was added and the plates placed on the orbital shaker at 900 rpm for 30 minutes. The plate then underwent a final wash procedure, and the remaining particles were then resuspended in wash buffer, placed on the orbital shaker at 900rpm for 5 mins, and read on a Bio-rad Bio-plex reader to determine individual cytokine concentrations interpolated from a standard curve of known concentrations.

**Supplementary Table 1.** Measured analytes with luminex and range of detection are listed in below

| **Measured Analytes** | ***Detection Range (pg/mL)*** |
| --- | --- |
| IL-6 | 1,460 - 2.0 |
| IL-8 | 1,440 - 2.0 |
| TNFα | 4,100 - 5.6 |
| LPS binding protein | 32,990,000 - 45,254 |
| Pro calcitonin | 4,160 - 5.7 |
| Soluble CD14 | 11,344,000 - 15,561 |
| LPS binding protein | 32,990,000 - 45,254 |

**Supplementary Table 2: Definitions of Extrahepatic Organ Dysfunction**

| **Definition of baseline organ dysfunction** | |
| --- | --- |
| Renal | Serum creatinine > 1.5md/dL |
| Cerebral | Grade III (Drowsy) or grade IV encephalopathy (coma) using the Westhaven Criteria to grade hepatic encephalopathy |
| Circulatory | Mean Arterial Pressure (MAP)* < 60mmHg or if the patient is receiving inotropic/vasopressor support |
| Respiratory | Sp0_2_/FiO_2_ of <357 |
| **Definition of new organ dysfunction** (Treatment D3 to end of treatment D15) | |
| Renal | Serum creatinine increase ≥50% compared to value at randomisation, rise in serum creatinine ≥0.3mg/dL within 48 hours or patient initiated on renal replacement therapy  *Note: patients receiving renal replacement at baseline could not reach this outcome* |
| Cerebral | Grade III (Drowsy) or grade IV encephalopathy (coma) using the Westhaven Criteria to grade hepatic encephalopathy  *Note: if the patient has grade III hepatic encephalopathy at baseline they will need to progress to grade IV to reach this endpoint* |
| Circulatory | i) MAP fall to <60mmHg OR  ii) patient is started on inotropic/vasopressor to support blood pressure  *Note: if MAP <60mmHg at baseline the inotropic/vasopressor support for blood pressure will need to be initiated to reach endpoint* |
| Respiratory | Any single point increase in Sp0_2_/Fi0_2_ as classified in the following scoring system as compared to Sp0_2_/Fi0_2_ at baseline:   \|  \| 0 \| 1 \| 2 \| \| --- \| --- \| --- \| --- \| \| Sp0_2_/Fi0_2_ \| >357 \| >214 to ≤357 \| ≤214 or mechanical ventilation for respiratory failure \| |

**Supplementary Table 3.** Mean difference and Boostrap Mean difference for matched and unmatched continuous propensity score baseline characteristics.

| Variable | Mean Difference | 95% CI | Bootstrap Mean Difference | Bootstrap 95% CI |
| --- | --- | --- | --- | --- |
| Unmatched |  |  |  |  |
| Age | -0.2967 | -2.2449 to 1.6515 | -0.29669 | -2.02857 to 1.57200 |
| MELD | -0.8809 | -2.0775 to 0.3158 | -0.88086 | -1.97257 to 0.31683 |
| Albumin | 0.3414 | -0.3050 to 0.9877 | 0.34138 | -0.30743 to 0.93093 |
| Creatinine | 11.5512 | 0.2703 to 22.8321 | 11.5512 | 0.5243 to 22.3702 |
| WCC | -2.3010 | -3.0502 to -1.5518 | -2.30102 | -2.98544 to -1.54045 |
| CRP | -2.7051 | -16.9451 to 11.5349 | -2.70507 | -13.6475 to 13.4334 |
| Matched |  |  |  |  |
| Age | -1.7990 | -4.4017 to 0.8037 | -1.79903 | -4.35601 to 0.70989 |
| MELD | 0.6089 | -0.7251 to 1.9429 | 0.60887 | -0.72546 to 1.86774 |
| Albumin | -0.0476 | -0.9723 to 0.8771 | 0.047619 | -0.98413 to 0.88889 |
| Creatinine | 4.0794 | -7.2819 to 15.4406 | 4.07937 | -6.7381 to 15.6032 |
| WCC | 0.1254 | -0.7319 to 0.9827 | 0.12540 | -0.71984 to 0.98254 |
| CRP | 6.9428 | -9.0098 to 22.8954 | 6.94281 | -6.0491 to 24.3371 |

**Supplementary Table 4.** Propensity score variables. * denotes an interaction between the variables.

| **Parameter Estimates** | | | | | | | | |
| --- | --- | --- | --- | --- | --- | --- | --- | --- |
| **Effect** | **Estimate** | **Standard Error** | **DF** | **t Value** | **Pr > \|t\|** | **Alpha** | **Lower** | **Upper** |
| **Intercept** | -0.5416 | 0.4688 | 743 | -1.16 | 0.2483 | 0.05 | -1.4619 | 0.3787 |
| **treat_creat** | 0.003025 | 0.003682 | 743 | 0.82 | 0.4116 | 0.05 | -0.00420 | 0.01025 |
| **treat_wcc** | -0.1307 | 0.04674 | 743 | -2.80 | 0.0053 | 0.05 | -0.2225 | -0.03896 |
| **treat_crea*treat_wcc** | 0.000169 | 0.000398 | 743 | 0.42 | 0.6720 | 0.05 | -0.00061 | 0.000950 |
| **treat_adm_vbld** | 0.3025 | 0.2514 | 743 | 1.20 | 0.2292 | 0.05 | -0.1910 | 0.7959 |
| **MELD0** | -0.01910 | 0.01794 | 743 | -1.06 | 0.2874 | 0.05 | -0.05431 | 0.01612 |

**Supplementary Table 5.** A comparison of baseline characteristics and mortality during trial treatment period between patients diagnosed with infection or not at trial entry, *denotes significant difference *p*<0.05. Mean and standard deviation (SD) presented.

|  | **Patients diagnosed with infection** | **SD** | **Patients not diagnosed with infection** | **SD** | ***P* value** |
| --- | --- | --- | --- | --- | --- |
| Number | 211 |  | 564 |  |  |
| Mean age (yrs) with SD | 55.1 | 10.4 | 53.3 | 10.6 | 0.35 |
| MELD Score | 19.8 | 5.8 | 19.69 | 6.3 | 0.76 |
| Creatinine (mmol/L) | 92.5 | 63.5 | 79.9 | 55.7 | 0.001 |
| WCC (x10^9^/L) | 10.5 | 6.2 | 8.2 | 4.6 | <0.0001 |
| CRP (mg/L) | 64.8 | 77.5 | 28 | 25.7 | <0.0001 |
| Deaths during trial | 29 (13.7%) |  | 37 (6.65) |  | 0.001 |

**Supplementary Table 6.** A Comparison of Baseline Characteristics and Clinical Outcomes for Patients taking either Carvedilol or Propranolol at ATTIRE trial entry. Mean and standard deviation (SD) presented.

|  | **Carvedilol** | **%/SD** | **Propranolol** | **%/SD** | ***P* value** |
| --- | --- | --- | --- | --- | --- |
| Number | 69 |  | 70 |  | n/a |
| Mean age (yrs) with SD | 54.4 | 8.3 | 52.8 | 10.3 | 0.32 |
| Male | 53 | 76.8% | 48 | 68.6% | 0.28 |
| Suspected Variceal Bleed | 18 | 26.1% | 11 | 15.7% | 0.13 |
| Ascites | 42 | 60.9% | 52 | 74.3% | 0.09 |
| Hepatic Encephalopathy | 11 | 15.9% | 11 | 15.7% | 0.97 |
| Diagnosis of infection at randomization | 11 | 15.9 | 14 | 20.0 | 0.53 |
| Baseline antibiotic use | 33 | 47.8% | 34 | 48.6% | 0.93 |
| MELD Score | 18.3 | 7.4 | 17.8 | 5.4 | 0.54 |
| Serum Albumin (g/L) | 24 | 2.9 | 24 | 3.9 | 0.56 |
| Creatinine (mmol/L) | 75 | 69.5 | 69.5 | 52.2 | 0.44 |
| WCC (x10^9^/L) | 6 | 3.8 | 6.3 | 3.7 | 0.42 |
| CRP (mg/L) | 18 | 29.5 | 19 | 103.3 | 0.23 |
| **Clinical Outcomes** | | | | | |
| Incidence of new infection | 11 | 15.9% | 11 | 15.7% | 0.97 |
| Incidence of Kidney dysfunction | 4 | 5.8% | 7 | 10.0% | 0.36 |
| 28-day mortality | 7 | 10.1% | 10 | 14.3% | 0.46 |
| 90-day mortality | 12 | 17.4% | 14 | 20.0% | 0.69 |
| 180-day mortality | 22 | 31.9% | 19 | 27.1% | 0.54 |

**Supplementary Table 7.** Baseline Characteristics and Clinical Outcomes for Patients with NSBB stopped within 5 days of trial entry, *denotes significant difference *p*<0.05. Mean and standard deviation (SD) presented.

|  | **NSBBs stopped**  **within 5 days** | **%/SD** | **NSBBs continued** | **%/SD** | ***P* value** |
| --- | --- | --- | --- | --- | --- |
| Number | 29 |  | 110 |  |  |
| Mean age (yrs) with SD | 52.8 | 9.8 | 53.8 | 9.3 | 0.59 |
| Male | 21 | 72.4% | 80 | 72.7% | 0.97 |
| Suspected Variceal Bleed | 9 | 31% | 20 | 18.2% | 0.13 |
| Ascites | 20 | 69% | 74 | 67.3% | 0.86 |
| Hepatic Encephalopathy | 4 | 13.8% | 18 | 16.4% | 0.74 |
| Diagnosis of infection at randomization | 3 | 10.3% | 22 | 20.0% | 0.23 |
| Baseline antibiotic use | 13 | 44.8% | 54 | 49.1 | 0.68 |
| MELD Score | 20.0 | 6.1 | 18.72 | 6.6 | 0.15 |
| Serum Albumin (g/L) | 23 | 3.5 | 23.5 | 3.5 | 0.53 |
| Creatinine (mmol/L) | 81.9 | 49.0 | 95.8 | 64.4 | 0.13 |
| WCC (x10^9^/L) | 7.4 | 3.7 | 6.8 | 3.7 | 0.27 |
| CRP (mg/L) | 28.4 | 34.5 | 29.9 | 33.7 | 0.53 |
| **Clinical Outcomes** | | | | | |
| Incidence of new infection | 2 | 6.9% | 20 | 18.2% | 0.14 |
| Incidence of new renal dysfunction | 4 | 13.8% | 7 | 6.4% | 0.19 |
| 28-day mortality | 7 | 24.1% | 10 | 9.1% | 0.03* |
| 90-day mortality | 8 | 27.6% | 18 | 16.4% | 0.17 |
| 180-day mortality | 10 | 34.5% | 31 | 28.2% | 0.51 |
