# Supplementary material for: Use of non-selective B-blockers is safe in hospitalised decompensated cirrhosis patients and exerts a potential anti-inflammatory effect: Data from the ATTIRE trial
Source: eClinicalMedicine. 2022 Nov 14;55:101716. doi: 10.1016/j.eclinm.2022.101716 (PMC9672423; doi:10.1016/j.eclinm.2022.101716)
Supplement: Supplementary File S2 [file mmc2.docx]

**Statistical Analysis Plan****: Evaluating the use of B-Blockers in patients admitted to hospital with decompensated cirrhosis: lessons from the ATTIRE trial**

***Justification:***

Use of non-selective Beta-Blockers (NSBBs) are well-established to reduce variceal bleeding. But in patients with cirrhosis with ascites, there are several unknowns:

- - - Does use increase incidence of renal dysfunction in patients with ascites?
    - Does use reduce mortality risk in patients with ascites?
    - Does use reduce the incidence of infection in patients with ascites?
    - Which is the best NSBB, Carvedilol or Propranolol: carvedilol has intrinsic anti-alpha adrenergic vasodilatory effects that contribute to its greater portal pressure reducing effect, but does this translate into improvement in clinical outcomes?

***Hypothesis:***

- In the ATTIRE trial cohort (Albumin to prevent infection in chronic liver failure), the use of NSBBs at baseline will reduce the risk of infection and improve mortality in patients hospitalised with decompensated cirrhosis compared to patients not taking NSBBs at baseline.

***Source of data for analysis:***

ATTIRE was a neutral trial of targeted albumin infusions versus standard care involving 777 hospitalized decompensated cirrhosis patients from 35 hospitals across England, Wales and Scotland (2016-2019). Data were collected daily until discharge, death, medically fit for discharge or day 15 and mortality data up to 6 months from trial entry. Data was inputted into the ATTIRE database at UCL Comprehensive Clinical Trials Unit. Data were collected daily until discharge, death, medically fit for discharge or day 15 and mortality data at 28-days, 3 and 6-months from trial entry.

ATTIRE defined renal dysfunction during trial treatment period, defined as serum creatinine increase ≥50% from randomisation, or patient initiated on renal replacement, or rise in creatinine ≥26.5 μmol/L within 48 hours. Daily incidence of respiratory, circulatory and cerebral dysfunction (grade 3 or 4 hepatic encephalopathy) during the treatment period (based on modified components of the Chronic liver failure-sequential organ failure assessment (CLIFSOFA) score) as well as pulse and blood pressure were recorded. Infection was according to attending clinician’s diagnosis and sites were then asked to complete infection CRFs with supporting clinical, biochemical, microbiological, and radiological data. These were blindly scrutinized by a panel of 3 physicians to categorize information provided as making infection diagnosis “likely” or “unlikely”. Blood test results were taken from values obtained at each hospital site.

B-blocker use will be extracted from the concomitant medication (ConMed) case report forms (CRFs). We will search for Propranolol, Carvedilol, Nadolol and Timolol.

***Ethical Approval:***

The ATTIRE trial was approved by the London–Brent Research Ethics Committee (ref:15/LO/ 0104) and the Medicines and Healthcare Products Regulatory Agency (MHRA, ref: 20363/0350/001-0001).

***Analyses:***

- Overall Patient Baseline characteristics: comparison of those taking and not taking NSBBs.

***Primary analyses to be performed:***

- To compare the effect of use of NSBBs at ATTIRE trial entry on renal and cardiovascular dysfunction in patients hospitalized with decompensated cirrhosis during hospitalisation on days 3-15 of trial (trial treatment period).
- To compare effect of NSBBs on mortality at 28 days, 3 and 6 months.

***Secondary analyses to be performed:***

- To compare the effect of NSBBs at ATTIRE trial entry on baseline infection diagnosis and systemic inflammation (white cell count, and C-reactive protein) and baseline plasma markers of bacterial translocation (endotoxin binding protein and soluble CD14), systemic inflammation (Tumour Necrosis Factor and Interleukin-6) and infection (procalcitonin and the neutrophil associated chemokine Interleukin-8) pre-albumin treatment.
- To compare hospital acquired infections, brain and respiratory dysfunction on days 3-15 of the trial between patients prescribed NSBBs or not at baseline (trial entry).
- To compare clinical outcomes on days 3-15 of hospitalization (infection; renal and cardiovascular dysfunction) ; and mortality on days 28, 3 and 6 months in patients taking either carvedilol or propranolol at baseline, and in patients with NSBBs stopped within 5 days of randomisation compared to those in which NSBBs were continued.

***Statistical analysis:***

- T-tests to be used for continuous variables and Fishers exact or Chi-squared tests for categorical variables.
- Confidence intervals are not adjusted for multiple comparisons.
- Propensity score matching to be used to account for baseline differences in disease severity, when numbers adequate to provide a robust basis for estimation, which we have defined prospectively as >50.
- Propensity Scoring to further examine use of NSBBs at ATTIRE trial entry on renal and cardiovascular dysfunction in patients hospitalized with decompensated cirrhosis: This was used to account for baseline differences in disease severity, when numbers were adequate to provide a robust basis for estimation, defined prospectively as >50. We plan a propensity score for each subject, using the fitted value on the logit scale from a logistic regression model which includes baseline use of antibiotics, suspected variceal bleed, new-onset/worsening ascites, hepatic encephalopathy, gender, age, MELD score, serum albumin, creatinine, white cell count (WCC), C-reactive protein (CRP), and randomized group. Explanatory variables in the baseline model will be modified until an adequate match is achieved between cases and controls assessed by relevant standardized mean differences. Cases and controls were matched on propensity scores using a ‘greedy’ nearest neighbor matching procedure without replacement, with caliper width of .01 on the logit scale. Once adequate matching has been been achieved, the matched data set will be locked locked before proceeding to outcome analyses.

***Software:***

Microsoft Excel to be used for extraction of data from ATTIRE databases, producing tables and graphs. IBM SPSS – Version 27 to be used for bivariate tests and SAS software, version 9.4 (SAS Institute; Carey NC) to be used for other analyses.
